# An Immune Basis for Malaria Protection by the Sickle Cell Trait

**DOI:** 10.1371/journal.pmed.0020128

**Published:** 2005-05-31

**Authors:** Thomas N Williams, Tabitha W Mwangi, David J Roberts, Neal D Alexander, David J Weatherall, Sammy Wambua, Moses Kortok, Robert W Snow, Kevin Marsh

**Affiliations:** **1**Kenya Medical Research Institute/Wellcome Trust Programme, Centre for Geographic Medicine ResearchCoast, Kilifi District Hospital, KilifiKenya; **2**Nuffield Department of Medicine, John Radcliffe HospitalOxfordUnited Kingdom; **3**Department of Paediatrics, John Radcliffe HospitalOxfordUnited Kingdom; **4**Blood Research Laboratory, National Blood Service—OxfordJohn Radcliffe Hospital, OxfordUnited Kingdom; **5**Nuffield Department of Clinical Laboratory Sciences, John Radcliffe HospitalOxfordUnited Kingdom; **6**Medical Research Council Tropical Epidemiology Group, London School of Hygiene and Tropical MedicineLondonUnited Kingdom; **7**Weatherall Institute of Molecular Medicine, John Radcliffe HospitalOxfordUnited Kingdom; University of RomeItaly

## Abstract

**Background:**

Malaria resistance by the sickle cell trait (genotype HbAS) has served as the prime example of genetic selection for over half a century. Nevertheless, the mechanism of this resistance remains the subject of considerable debate. While it probably involves innate factors such as the reduced ability of Plasmodium falciparum parasites to grow and multiply in HbAS erythrocytes, recent observations suggest that it might also involve the accelerated acquisition of malaria-specific immunity.

**Methods and Findings:**

We studied the age-specific protection afforded by HbAS against clinical malaria in children living on the coast of Kenya. We found that protection increased with age from only 20% in the first 2 y of life to a maximum of 56% by the age of 10 y, returning thereafter to 30% in participants greater than 10 y old.

**Conclusions:**

Our observations suggest that malaria protection by HbAS involves the enhancement of not only innate but also of acquired immunity to the parasite. A better understanding of the underlying mechanisms might yield important insights into both these processes.

## Abstract 2

In a Kenyan population, protection against malaria by sickle cell trait increased over the first 10 years of life, suggesting that the mechanism of protection involves acquired immunity to the parasite.

## Introduction

Sickle cell trait (genotype HbAS) confers a high degree of resistance to severe and complicated malaria [1–4] yet the precise mechanism remains unknown. To some extent it almost certainly relates to the peculiar physical or biochemical properties of HbAS red blood cells: invasion, growth, and development of Plasmodium falciparum parasites are all reduced in such cells under physiological conditions in vitro [5,6], and parasite-infected HbAS red blood cells also tend to sickle [5,7,8], a process that may result in their premature destruction by the spleen [5,9]. Nevertheless, while such factors appear to be important, recent observations suggest that the mechanism might also involve an immune component. For example, in a study conducted in Gambia, we found that the immune recognition of P. falciparum–infected red blood cells was enhanced in HbAS children [10], and up-regulation of malaria-specific cell-mediated immune responses has also been observed in HbAS individuals in Sudan [11,12]. While potentially important, such observations could represent epi-phenomena, rather than proximate effects of the HbAS red cell phenotype. Establishing whether or not immune processes are involved may prove useful in learning about malaria protection more generally. We have therefore investigated this question by studying the age-specific pattern of malaria disease in children living on the coast of Kenya. We reasoned that if malaria protection by HbAS was predominantly innate, it should be independent of malaria exposure and therefore remain constant with age. Conversely, if immune mechanisms were involved, the degree of protection should increase with age up until the age when children generally become functionally immune to malaria, at which time any additional immunological advantage should be lost.

## Methods

### Patients and Methods

The study was conducted in a cohort of children and adults living within the Ngerenya and Chonyi areas of Kilifi District on the coast of Kenya as described in detail previously [13,14]. Briefly, participants were recruited from an age-stratified population sample weighted towards children less than 10 y old, and between September 1998 and March 2004 study participants were monitored by active surveillance for clinical events with a focus on malaria. Children born into study households during the course of the program were recruited at birth, and participants exited from the study if informed consent was withdrawn, if they moved out of the study area for more than 2 mo, or if they died. Hemoglobin types were available for 1,054 of 1,795 total cohort members who attended periodic cross-sectional surveys conducted throughout the study. We have previously defined malaria as a fever (axillary temperature >37.5 °C) in association with malaria parasitaemia of any density in children less than 1 y old or at a density of greater than 2,500 parasites/μl in older children [13]. However, HbAS has a significant effect on the densities of incident malaria infections, and we have no data that allow us to confirm whether or not this definition is also appropriate for such children. For the purposes of this analysis, we have therefore used a conservative definition of malaria—fever in association with a slide positive for blood stage asexual P. falciparum parasites at any density. Incident malaria infections were treated with sulphadoxine–pyrimethamine according to local guidelines.

### Laboratory Procedures and Statistical Analysis

Blood films were stained and examined for malaria parasites by standard methods, and haemoglobin types were characterized by electrophoresis. We compared the incidence of malaria in HbAS individuals versus individuals without the sickle cell allele (genotype HbAA) by Poisson regression (with malaria as the dependent variable) both with and without adjustments for the following confounding variables: season (defined as 90-d blocks), study area (Ngerenya or Chonyi), ethnic group, and age (in 2-y bands until the age of 10 y, older participants being classified in the top band as described in Table 1). Participants were considered not to be at risk of malaria and were dropped from both numerator and denominator populations for 21 d after receiving treatment with an anti-malarial drug. Because all infants are relatively resistant to malaria during the first 3 mo of life [15], we excluded children less than 3 mo old from our analyses. Because the study was conducted over a prolonged period, most participants contributed data to more than one age stratum. We took account of potential within-person clustering of malaria events, both within and between age strata, by using the “sandwich” estimator as described by Armitage and colleagues [16], which inflates confidence intervals and adjusts significance values as appropriate. We have expressed our comparisons as adjusted incidence rate ratios (IRRs). We investigated the possibility that age might be acting as an effect modifier in the association between malaria and haemoglobin type by comparing models that included or excluded interaction terms between haemoglobin type and age using the Wald test. All analyses were conducted using STATA version 8.0 (StataCorp, Timberlake, London, United Kingdom).

**Table 1:**
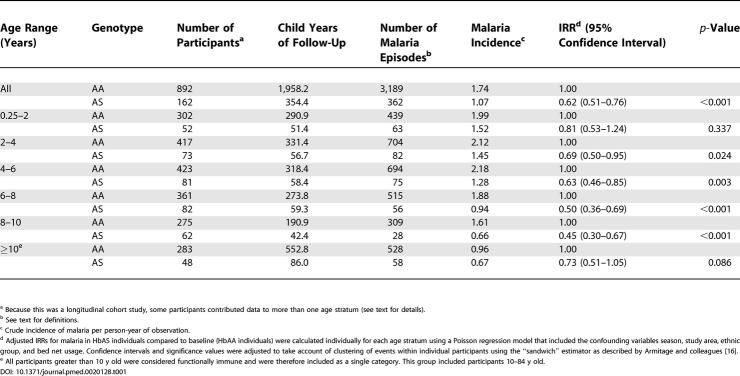
Incidence of Clinical Malaria by Age and Haemoglobin Type

^a^ Because this was a longitudinal cohort study, some participants contributed data to more than one age stratum (see text for details).

^b^ See text for definitions.

^c^ Crude incidence of malaria per person-year of observation.

^d^ Adjusted IRRs for malaria in HbAS individuals compared to baseline (HbAA individuals) were calculated individually for each age stratum using a Poisson regression model that included the confounding variables season, study area, ethnic group, and bed net usage. Confidence intervals and significance values were adjusted to take account of clustering of events within individual participants using the “sandwich” estimator as described by Armitage and colleagues [16].

^e^ All participants greater than 10 y old were considered functionally immune and were therefore included as a single category. This group included participants 10–84 y old.

Ethical permission for the study was granted by the Kenya Medical Research Institute National Ethical Review Committee. Individual written informed consent was provided by all study participants or their parents.

## Results

**Figure 1:**
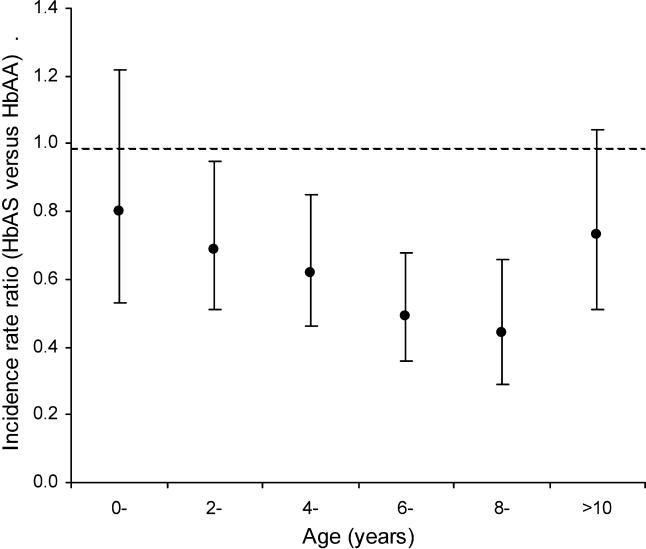
The IRR for Malaria in HbAS versus HbAA Children by Age and Genotypic Group Infants less than 3 mo old were excluded from the baseline group.

Overall, HbAS was almost 40% protective against mild clinical malaria (IRR = 0.62; 95% confidence interval 0.51–0.76; *p* < 0.001); however, protection appeared to vary with age, increasing from only 20% to almost 60% over the first 10 y of life and returning to around 30% thereafter (Table 1; Figure 1). A similar pattern was seen when data from each of the study areas were analyzed separately. Although we were not able to prove statistically an overall interaction between age and protection over the full range of ages (χ^2^_5_ = 6.46; *p* = 0.26), the data support the strong impression of acquired protection with age.

## Discussion

The mechanism by which HbAS protects against malaria has been the subject of speculation for more than 50 y. While to some extent it probably relates to the physical characteristics of HbAS erythrocytes, a number of studies suggest that HbAS may also enhance the acquisition of natural immunity [10,17–19]; however, establishing this relationship is difficult because immunity to malaria is hard to measure.

To date, no single immune response has been described that reliably predicts protective immunity. As a result, immunity to malaria is usually defined as the ability to control new infections to a level at which they fail to reach a clinical threshold. We therefore reasoned that the best way to find out whether malaria protection by HbAS involves a significant immune component was to see whether protection varies with age. Of the cohort studies that have been reported to date, most have involved repeated cross-sectional sampling rather than active monitoring for clinical events. Moreover, of the studies that have investigated the genotype-specific incidence of mild malaria [10,20–23], all have been either too small, have involved a restricted age range of participants, or have been conducted over too short a period to make it possible to address this important question. Our study is, to our knowledge, the first with sufficient power to observe the protective effect of HbAS over a broad age range. We found that HbAS protection increases throughout the first 10 y of life, returning thereafter to baseline. While it is possible that this observation could result from any factor that both affects malaria risk and varies with age, accelerated immune acquisition seems by far the most likely explanation.

So how might HbAS result in the accelerated acquisition of malaria-specific immunity? A number of mechanisms have been proposed. In common with other red cell genetic defects, enhanced phagocytosis of HbAS erythrocytes infected with ring stage P. falciparum has been demonstrated in vitro [24], a process that appears to be mediated by a mechanism essentially similar to that involved in the phagocytosis of senescent or damaged normal erythrocytes. Experimental data suggest that this process is initiated by enhanced oxidant damage to the erythrocyte membrane and that this leads to the aggregation of band 3 protein and the binding of autologous IgG and complement [24], a mechanism similar to that previously proposed for α thalassaemia [25]. It therefore seems plausible that enhanced immunity could be mediated by the accelerated acquisition of antibodies to altered host antigens expressed on the parasite-infected red cell surface, such as band 3 protein [26]. On the other hand, parasite-derived proteins such as the variant surface antigen P. falciparum erythrocyte membrane protein-1 might represent an alternative target, an hypothesis supported by the raised titres of antibodies directed towards variant antigens seen in HbAS children living in Gambia [10]. These mechanisms need not be mutually exclusive.

As an alternative explanation, it seems possible that by controlling parasite densities during malaria infections [27] innate processes might paradoxically increase the chronicity of individual infections. This hypothesis is supported by the greater number of strains of P. falciparum parasites found in HbAS than HbAA children at cross-sectional survey [28]. By increasing the duration of individual malaria infections HbAS might paradoxically increase host exposure to a variety of antigens capable of inducing malaria-specific immunity. Determining which if any of these mechanisms are involved could lead to a better understanding of malaria immunity more generally.

In our current study we have focused on mild clinical malaria. For accelerated malaria-specific immunity to be relevant to HbAS selection it would have to operate within the period of maximum risk for severe and fatal malaria. In Kilifi, this risk is greatest in children less than 5 y old [29]. It is clear from a recent study conducted in western Kenya [4] that HbAS is strongly protective against severe and fatal malaria within this age range; however, protection by HbAS against both severe malaria anaemia and all-cause mortality was only seen in the age range 2–16 mo. The authors suggested that this may have reflected early protection by maternally transferred immunoglobulins followed by the general acquisition of protective immunity after the age of 16 mo; however, they presented no data regarding the effect of age between these extremes. Given the level of protection conferred by HbAS against severe malaria, it is possible that their study was not sufficiently powered to address this question. The relevance of our observations in mild clinical malaria to the protection afforded by HbAS against severe and fatal malaria therefore remains unknown. While immunity against severe malaria develops significantly more rapidly than immunity to mild clinical attacks, the determinants of each remain poorly understood. We suggest that establishing the role of HbAS in each of these processes may be one route to learning more about the mechanisms involved.

**Patient Summary**

**Background**

Sickle cell anemia, which is caused by having two copies of an abnormal gene (hemoglobin S—HbS) that causes red cells to deform easily, occurs more frequently in populations exposed to malaria. Previous work has shown that carrying one normal copy of the gene (HbA) and one copy of the version responsible for sickle cell disease (the combination is called HbAS) may protect against getting malaria; hence, this abnormal gene provides an advantage to some people who carry it. How this protection happens is unclear, but may be due to changes in the way that people with HbAS develop immunity to malaria.

**What Did the Authors Do?**

One way of working out whether acquired immunity is important in how HbAS protects against malaria is to look at a large population with many different age ranges, all exposed to malaria, and measure how often these individuals get malaria. The authors of this paper looked at 1,054 people in Kenya with an age range from birth up to 84 years, but predominantly aged less than 10 years, who either had HbAS or normal hemoglobin (HbAA). They found that protection of HbAS against mild malaria increased with age from 20% in the first two years of life to a maximum of 56% by the age of ten years, and then decreased to 30% in people older than ten years.

**What Do These Findings Mean?**

The presence of HbAS is associated with increased acquired immunity to mild malaria. Further work will need to be done to work out how this change in immunity occurs. It is not yet known whether these results are also true for protection against severe malaria, and in any case the protection is only partial; hence, treatment of anyone with malaria, whatever their sickle cell status, is essential.

**Where Can I Get More Information?**

The Centers for Disease Control and Prevention publish reviews of various conditions. The one on sickle cell disease includes links to other sources of information: http://www.cdc.gov/genomics/hugenet/reviews/sickle.htm

The World Health Organization has a Web page on malaria: http://www.who.int/topics/malaria/en/

## Abbreviations

IRR: incidence rate ratio
